# Health Care Professionals’ Barriers and Facilitators for the Implementation of Lifestyle Interventions Targeting Weight Loss in Primary Care – A Systematic Review of Qualitative Studies

**DOI:** 10.1007/s13679-026-00721-8

**Published:** 2026-06-04

**Authors:** Maxime A.M. Van der Velden, Priya Gharbaran, Nuria Jansen, Maxim de Jong, Patrick J.E. Bindels, Marienke Van Middelkoop

**Affiliations:** Department of General Practice, Erasmus MC Medical University Center Rotterdam, PO Box 2040, Room Na-19, Rotterdam, CA 3000 The Netherlands

**Keywords:** Barriers and facilitators, Lifestyle interventions, Implementation, Obesity, Primary care

## Abstract

**Purpose of Review:**

This systematic review aims to investigate barriers and facilitators that Health Care Professionals (HCPs) experience during the implementation of a lifestyle intervention for patients with overweight or obesity targeting weight loss in primary care.

**Recent Findings:**

Overweight and obesity are one of the most pressing public health problems worldwide and have severe health consequences. Lifestyle interventions are the first treatment in line for patients with overweight and obesity. HCPs are trained to provide lifestyle interventions, but implementing these interventions in primary care remains challenging as HCPs face multiple barriers. There is no clear overview of the perceived barriers and facilitators experienced by HCPs to implement lifestyle interventions targeting weight loss for patients with overweight or obesity in primary care.

**Summary:**

Main reported barriers by HCPs were lack of time, unavailability of staff, lack of tools and limited financial resources. Interventions fitting HCPs workflow, structural financial support, training and tools were reported as facilitators for successful implementation of the intervention. Policy makers should ensure future lifestyle interventions take these barriers and facilitators into account enhancing the implementation and delivery of the intervention by HCPs to their patients in practice.

**Supplementary Information:**

The online version contains supplementary material available at 10.1007/s13679-026-00721-8.

## Introduction

In 2030, it is expected that the majority of the world’s population will have either overweight or obesity [1, 2]. Overweight and obesity have negative effects on physical (e.g. diabetes, cardiovascular diseases, cancer) and mental health (e.g. low self-esteem, loneliness, depression) [3, 4]. It is therefore of great importance to address overweight and obesity to improve the overall health and quality of life of individuals struggling with their weight. In many countries primary care is the first point of care, and according to international primary guidelines primary health care providers (HCPs) should address overweight and obesity in their patients. HCPs therefor play a pivotal role in the management of overweight and obesity [5].

A wide range of treatments for patients with overweight and obesity to lose weight are available, such as lifestyle, pharmacological and bariatric surgical interventions [3, 6]. Although the upcoming weight loss medications against overweight and obesity are very effective, lifestyle interventions are still the first treatment in line for patients with overweight and obesity since these are least invasive and have promising results on health outcomes on the long-term [7–9]. Lifestyle interventions are often multicomponent programs with the aim to lose weight by implementing changes in behavior including diet and physical activity [10, 11]. Despite a positive effect of lifestyle interventions on weight loss and improved health status of patients is observed, challenges such as high dropouts, low patient motivation and commitment are often reported [6, 12, 13].

As HCPs are trained to provide health care, they are seen as a reliable reference to give health advice and to guide their patients [14, 15]. Furthermore, patients appreciate the trusting relationship with HCPs and regard their expertise [16]. However, delivering lifestyle interventions in primary care remains challenging due to shortness of time, lack of skills of HCPs or unclarity about insurance coverage for patients [6]. Although multiple studies have investigated the perspectives of HCPs on lifestyle interventions, there is no clear overview of the perceived barriers and facilitators for HCPs to implement lifestyle interventions for patients with overweight or obesity targeting weight loss in the primary care setting.

Therefore, the purpose of this systematic review is to synthesize the literature on perceived barriers and facilitators experienced by HCPs for the implementation of lifestyle interventions for patients with overweight and obesity targeting weight loss in primary care. The outcomes of this review will provide useful insights to improve the implementation of lifestyle interventions in primary care in the future.

## Method

This systematic review was conducted and reported in line with the PRISMA statements [17]. The protocol for the review (ID CRD42023471913) was registered before initiating data extraction on November 8, 2023 and can be found at crd.york.ac.uk/PROSPERO/display_record.php? ID=CRD42023471913.

### Search Strategy

With the support of the Erasmus MC Medical Library, a search strategy was developed to identify studies directed at barriers and facilitators perceived by HCPs for implementing lifestyle interventions targeting weight loss in primary care. Four databases (Embase, MEDLINE, CINAHL EBSCO, and Web of Science SCIE & SSCI) were searched on November 22, 2024 using keywords such as: general practice, lifestyle, body weight management, overweight, obesity, barriers, facilitators, patient attitude (see Appendix S1 for the full search strategy).

### Eligibility Criteria

#### Study Design

Qualitative studies with outcomes from interviews (including both individual interviews and focus groups), field notes and questionnaires were included. Quantitative outcomes were not included.

#### Participants

The following HCPs were included: general practitioners (GPs), family doctors, pediatricians, practice nurses, practice assistants, lifestyle coaches, dieticians and physiotherapists. The HCPs must be working in primary care and should be involved in the treatment of adults and/or children with overweight or obesity. Interventions for children (aged 5–12), adolescents (aged 13–18) and adults (aged > 18) were included. Overweight and obesity were defined according to the criteria of the World Health Organization (WHO) [18]. For adults (aged > 19), overweight was classified as BMI ≥ 25 and obesity as BMI ≥ 30. For children (aged 5–18), a BMI of > 1 standard deviation (SD) above the median is considered overweight and a BMI of > 2 SD above the median is considered obese. Lifestyle interventions focusing on participants with comorbidities were allowed, except for pregnant women participating in lifestyle interventions related to their pregnancy and people with mental health issues.

#### Intervention

Studies had to be performed in primary care and directed at lifestyle interventions with the aim of losing weight. Dietary intake, physical activity, eating behavior, psychological guidance, or a combination were deemed to be lifestyle interventions and were therefore included. Surgical or pharmacological interventions, or interventions combining lifestyle interventions with surgery or medications were excluded.

#### Outcome

Studies needed to assess the barriers and facilitators perceived by HCPs towards the implementation of lifestyle interventions for patients with overweight or obesity in primary care. Barriers were defined as reasons that make it more difficult for HCPs to implement a lifestyle intervention, whereas facilitators were defined as reasons that make it less difficult for HCPs to implement a lifestyle intervention. Studies without a proposed intervention were not included, as the barriers and facilitators reported are not applicable in this review. Studies reporting HCPs barriers and facilitators regarding implementation of lifestyle interventions in general, without mentioning a proposed intervention were also not included, as this is not a specific lifestyle intervention.

### Selection of Studies

For the selection of eligible studies, a double-screening approach was utilized where two researchers (MV and PG) independently assessed all studies of each other. First, titles and abstracts of each study were screened by MV and PG. Articles written in English and Dutch were included and abstracts for congresses or duplicates were removed. Second, the full-text of each study was independently read by MV and PG. For each included or excluded study a reason for was stated by MV and PG. Disagreements were resolved by discussion.

### Quality Assessment

To assess the quality of each included study, the Qualitative Studies Checklist from the Critical Appraisal Skills Program (CASP) [19] was used (see Appendix S2 for the checklist). This checklist consists of ten different domains, each accompanied by questions to assist in the assessment of the quality. The questions marked as highly important are listed in Appendix S2. Two researchers (MV, PG) independently assigned a score of 0 or 1 per domain to each study; to obtain a score of 1, the most important questions had to be answered positively. Disagreements were resolved through discussion and by consulting a third author (MM). An overall score ranging from 0 to 10 was calculated from the 10 domains, with higher scores indicating higher study quality.

### Data Extraction and Synthesis

Study characteristics were extracted and checked by MM and PG following a standardized format including setting, study aim, lifestyle intervention, inclusion and exclusion criteria, type of HCPs, data collection method and relevant outcomes.

Thematic synthesis was used to find key themes across the various studies [20]. Firstly, all the verbatim results of the studies were entered into MaxQDA and initial codes were assigned. Using MaxQDA connections were then made by MM and PG between the different codes to group them into overarching themes. In the final step, major analytical themes were constructed to generate new insights into the implementation of the lifestyle interventions and to obtain a better understanding of the most common barriers and facilitators.

## Results

**Fig. 1:**
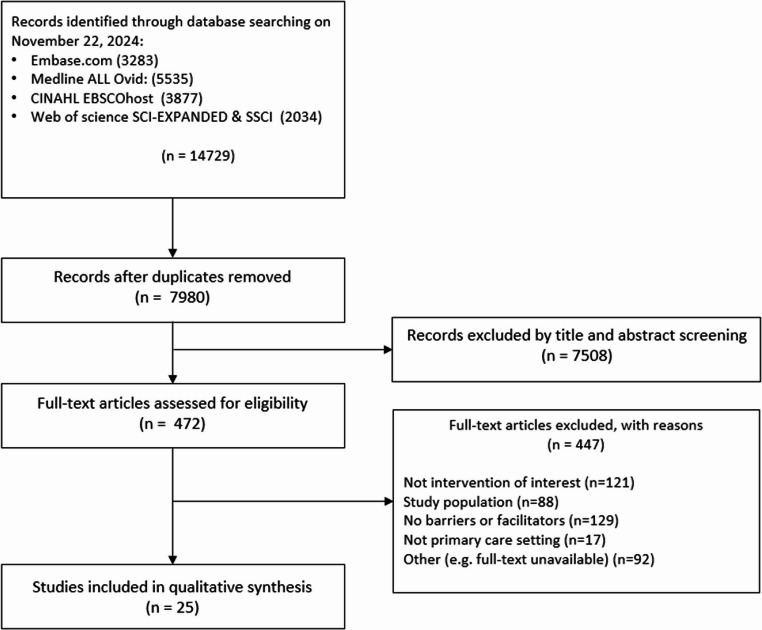
Flowchart for the screening and selection of studies

The search strategy identified 7980 records which were first screened by abstract and title; 472 articles were included for full-text screening, of which 25 articles were included (Fig. 1).

### Study Characteristics

The study characteristics of the 25 included studies can be found in Table 1 (see Appendix S3 for additional information).

**Table 1:** Study characteristics of the articles included

	Author + Year	Setting (Country)	Lifestyle intervention	Inclusion criteria HCPs	*N* in qualitative part	Type of HCPs	Sex participants: women %	Participants: Average age in years	Data collection	Relevant outcomes reported in results
1	Alsaeed et al., 2022 [6]	Primary care(Kuwait).	Total Dietary Replacement (TDR) intervention utilizes formula low-energy diet meal replacement products for a period of 12 weeks, followed by a 12 weeks structured food reintroduction phase with behavioural support.	Experience providing weight loss advice and directly involved with patients with type 2 diabetes.	17	Dietitians	17 (100%)	30.8	Focus group interviews (*n* = 3)	Perceived barriers of TDR and suggestions to improve approach. The three emerging themes were motivation to use the TDR approach, perceived challenges of TDR, and suggestions to improve and adapt approaches.
2	Ayyaswami et al., 2024 [42]	Primary care (USA)	A clinician and electronic health record (EHR) wearable device intervention to increase physical activity (PA) in patients with obesity.	Physician, residents, physician, or advanced practitioner provider practicing in the UMass Memorial Medical Group Primary Care Clinic.	12	Primary care providers	6 (50%)	Not reported	Semi-structured interviews	Implementation of an EHR-based RPM program and associated workflow is acceptable to PCPs. Three themes were identified from provider interviews: (1) providers knowledge of PA prescription and limited knowledge on hot wo tailor guidance to patients, (2) worried about being overburdened by additional patient data, (3) concerns about patients ability to equitably access and participate in digital health interventions.
3	Bennett et al., 2014 [26]	Primary care (USA)	Practice-based Opportunities for Weight Reduction (POWER)-Trial: structured behavioral weight loss intervention for obese adults to lose weight more effectively than standard care.	Not reported	26	Physicians (*n* = 24) and nurses (*n* = 2)	15 (57.7%)	46	Semi-structured focus group interviews	PCPs roles in weight management, inclusive of and beyond the role intended by the trial’s design, and recommendations for wider dissemination of the POWER-trial its integration into primary care practice. PCPs provided several highlighted the need for specific feedback from coaches as well as efficient integrated processes.
4	Blane et al., 2017 [21]	Primary care (UK)	Weight management services	Not reported	9	Dieticians	7 (77.8%)	Not reported	Small group and individual interviews	Views of key stakeholders involved in the planning and delivery of adult weight management services on the role of primary care in adult weight management and their experience of engaging with GPs and practice nurses.
5	Brandt et al., 2018 [27]	Municipating primary care (Denmark)	eHealth coaching	Not reported	10	HCPs (dieticians *n* = 5; physiotherapists *n* = 2; nurses *n* = 1; occupational therapists *n* = 1; nurse assistant *n* = 1)	10 (100%)	48	Individual semi-structured interviews	Four themes were identified relevant to the health care professionals in their asynchronous eHealth coaching: (1) establishing an empathic relationship, (2) reflection in asynchronous eHealth coaching, (3) identifying realistic goals based on personal barriers, and (4) staying connected in asynchronous coaching.
6	Burton et al., 2023 [24]	Primary care (UK)	Transferability of the NHS low-calorie diet (LCD) programme.	Not reported	25	Locality lead *n* = 7; referrer *n* = 9; programme deliver *n* = 9	22 (88%)	Not reported	Semi-structured interviews	Stakeholders had confidence in the LCD programme due to the robust evidence base along with anecdotal evidence. Stakeholders described barriers to accessing the programme, including language and learning difficulties.
7	Chimoriya et al., 2023 [28]	Primary care (Australia)	A qualitative study of the perceptions and experiences of participants and healthcare professionals in the DiRECT-Australia type 2 diabetes remission service	Not reported	6	HCPs (GPs *n* = 3; practice nurses *n* = 2; dietician *n* = 1)	Not reported	55.4	Semi-structured interviews	Perceptions and experiences of healthcare professionals (HCPs) involved in the DiRECT- Australia Type 2 Diabetes Remission Service.
8	Cupit et al., 2021 [25]	Primary care (UK)	Low carb intervention: reduced carbohydrate diet	Not reported	19	HCPs (GPs *n* = 13; practice nurses *n* = 3; employed health coaches *n* = 1; ‘Volunteer Health Coaches’ n = 2)	9 (47.4%)	Not reported	Semi-structured interviews	Healthcare practitioner (HCP) experiences of implementing a reduced carbohydrate diet. Key themes highlight experiences of: (1) discovering low- carb as a new ‘tool- in- the- box’; (2) promoting and supporting incremental low- carb experimentation; and (3) diverging from established dietary guidelines.
9	Darling et al., 2023 [29]	Primary care (USA)	HCPs referring to lifestyle interventions	(1) Self-identified as primarily treating adolescents; (2) reported that at least one third of their patient population from a low-income background	11	Paediatric medical providers	9 (82.0%)	45.9	Semi-structured individual interviews	Provider perceptions of barriers and facilitators to weight management engagement for youth from low-income backgrounds. Factors affecting providers’ decision to refer to WM programs included health implications, perceived motivation of the patient and family as many families experience shame or guilt.
10	Drew et al., 2024 [30]	Primary care (UK)	Normalisation and equity of referral to the NHS Low Calorie Diet programme pilot	Health care staff with experience of patient referral to the LCD programme (referred to hereon in as ‘referrers’), were recruited equally across the first ten localities who undertook the programme pilot.	19	HCPs (Practice Nurses *n* = 10, General Practitioners *n* = 6, Clinical Pharmacists *n* = 2 or Advanced Nurse Practitioners *n* = 1)	14 (73.7%)	Not reported	Semi-structured interviews	Experiences of health care staff who have made a referral to the LCD programme, while identifying effective and equitable delivery of programme referrals, and their implementation into routine care. Inequalities remain a significant challenge, and the adoption of an equitable referral process, normalised at a service delivery level, has the capacity to contribute to the improvement of health inequalities.
11	Finn et al., 2024 [38]	Primary care (USA)	SmartMoves intervention.	Not reported	12	Primary care providers	Not reported	Not reported	Individual interviews	Lessons from the dissemination of SmartMoves: System- and organizational-level barriers impeded sustainment of an evidence-based IHBLT program. Adequate funding could enable sufficient staffing and training to promote fidelity to the intervention’s core functions and adaptation to fit local populations/context.
12	Govindasamy et al., 2023 [31]	Primary care (Australia)	Mental contrasting and implementation intentions (MCII)	(1) have T2DM; (2) identified as obese or overweight with a BMI between 25 and 40 (3) age between 40 and 70 years	4	Practice Nurses	4 (100%)	Not reported	Semi-structured interviews	Challenges PNs experienced implementing MCII: PNs found challenges related to cultural expectations and the requirement of patients to set and adhere to dietary change goals and behaviours. Obstacles were also encountered in delivering the intervention in a busy general practice setting.
13	Johnson et al., 2018 [39]	Primary care (UK)	The intervention “Eat Well Move More” (EWMM) combines healthy eating and cooking education with physical activity sessions. Tree service offers exist: a school programme (4–16 years), community programme (7–11 years) and one-to-one sessions (12–16 years)	Not reported	4	GPs	Not reported	Not reported	Telephone interviews	Barriers for GPs to refer children to EWMM. Three main barriers were highlighted: (1) parent engagement, (2) child autonomy, and (3) concerns over the National Child Measurement Programme letter.
14	Nederveld et al., 2021 [40]	Primary care (USA)	Intense Behavioural Therapy (IBT)	Not reported	75	Primary care providers (Physician *n* = 50; Advanced practice clinicians *n* = 2; Registered dietitian *n* = 8; Other type *n* = 15)	Not reported	Not reported	Individual interviews by phone	Experiences of providing obesity management among primary care clinicians and their team members involved with weight loss in primary care practice. Challenges identified: addressing obesity; complex multifactorial problem; billing and reimbursement.
15	K. Paine et al., 2023 [44]	Primary care (Australia)	The HeLP-GP intervention supported overweight and obese participants by providing a FPN-led tailored health check based on the 5As model of patient-centred care in order to improve patients diet, increase their level of physical activity, and improve their general health	(1) 40–74 years; (2) overweight or obese (BMI > 28); (3) weight and blood pressure recorded within previous 12 months; (4) access to smart phone or tablet device; (5) speak and read either English, Arabic, Chinese or Vietnamese	12	General practitioners (*n* = 8); practice nurses (*n* = 4)	4 (30.0%)	Not reported	Semi-structured individual interviews	It aimed to deepen our understanding of the study implementation and outcomes, as well as to provide practical insights to guide future interventions of this type. Three key themes: long-term trusting and supportive relationships (being ‘in it for the long haul’); initiating conversations and understanding motivations; and ensuring access to multi-modal weight management options that acknowledge differing levels of health literacy
16	Parker et al., 2024 [32]	Primary care (Australia)	HeLP-GP aims to help overweight or obese patients to make positive lifestyle changes while assessing the value, sustainability and scalability of the nurse-led intervention.	Not reported	78	Practice nurses	54 (69.2%)	22–65	Mixed-method including surveys and observations	Implementation of the HELP-GP trial through the lens of organisational readiness with emphasis on the role of the practice nurse. A lack of general ‘readiness’ inherent in the nursing role, particularly related to their capacity to complete intervention tasks and practice-level support to implement the intervention.
17	Persaud et al., 2022 [23]	Primary care and community settings (USA)	Pediatric weight management interventions (PWMI) for obesity children to reduce BMI. Healthy weight clinic (HWC) intervention versus Modified Healthy weight and Your Child (M-HWYC) intervention.	Child had BMI ≥ 85th percentile for age and gender.	26	Stakeholders (multi-sector stakeholders including paediatricians, dietitians, community health workers, behavioral health professionals, program managers, chief medical officers, local YMCA directors, state community health center representatives, national YMCA representatives, a Medicaid official, and a parent and patient who had participated in previous PWMIs)	Not reported	Not reported	Individual interviews by phone	Experienced barriers and facilitators of stakeholders for the implementation and dissemination of PWMI. Highlighted the importance of engaging multi sector stakeholders pre-implementation to ensure the components valued are included, ensuring the program minimizes barriers to participation, considering how staff training can improve implementation and how collected outcomes can inform sustainability and dissemination of PWMIs in primary care.
18	Poppe et al., 2018 [43]	Primary care (Belgium)	“MyPlan 1.0”, a computer-tailored eHealth intervention based on self-regulation. “MyPlan 1.0” targets PA as well as fruit and vegetable intake of adults visiting general practice	Not reported	15	GPs	7 (46.7%)	47.2	Individual interviews	Experiences of GPs implementing “MyPlan 1.0” into general practice: GPs experienced severe time restrictions in general practice. GPs also seemed to select those patients who they believed to be able to use (e.g., highly educated patients) and to benefit from the intervention (e.g., patients with overweight)
19	Porter et al., 2021 [33]	Primary care (USA)	Weight management program	Not reported	51	Primary care staff (physician, nurse, physican assistant, health coach, coordinator, other clinic staff)	39 (71.0%)	Not reported	Semi-structured focus group interviews	Participants agreed it is possible to implement a weight management program through primary care, but cited potential facilitation challenges such as costs, clinic resources, and individual patient barriers. More enthusiasm arose for a referral program with patient tracking. Program characteristics such as proven efficacy, individual tailoring, program accessibility, and patient feedback to the providers were desired.
20	Rehackova et al., 2022 [22]	Primary care (UK)	The Diabetes Remission Clinical Trial (DiRECT): 12–20 weeks of TDR (825–853 kcal per day, Counterweight PRO800 provided by Cambridge Weight Plan), followed by 6–8 weeks of FR, and structured support for WLM for 2 years.	Not reported	10	HCPs (practice nurses and dieticians)	Not reported	Not reported	Interviews by phone or face-to-face	Challenges and facilitators of implementation for HCPs, which would likely affect success of the intervention delivered at scale: concerns over perceived potential negative intervention effects was a barrier to engagement. Trust of HCPs towards the research team and perceived credibility of the study facilitated engagement and adoption. Ongoing support by research dietitians was key to the management of participants.
21	Simione et al., 2020 [34]	Primary care (USA)	Connect for Health is an evidence-based weight management program with clinical- and family-facing components for delivery in paediatric primary care for families of children ages 2 to 12 years	Not reported	52	HCPs (Physician *n* = 44; Medical assistant *n* = 4; Nurse practitioner *n* = 2; Physician’s assistant *n* = 2)	40 (76.9%)	Not reported	Individual interviews	Contextual barriers, facilitators, and organizational readiness for the uptake of the proposed program tools and implementation strategies
22	Smith et al., 2017 [35]	Primary care (UK)	POWeR+	Not reported	13	HCPs	13 (100%)	Not reported	Individual telephone interviews	Practitioners found POWeR+ straightforward and easy to use. Some practitioners preferred providing support face-to-face, which they enjoyed more than remote support. A small number of nurses found providing non-directive support using the CARe approach (Congratulate, Ask, Remind) challenging, feeling it was the opposite of their normal approach.
23	Thomas et al., 2022 [41]	Primary care (Sweden)	The mHealth intervention “MINISTOP”: a mobile application that was initially developed targeting parents of 4-year-olds, aimed to reduce the prevalence of overweight and obesity by giving support to improve diet and physical activity.	(1) currently employed at one of the participating centers and (2) willing to participate	15	Practice Nurses	Not reported	47 years	Semi-structured interviews by phone	Barriers for implementation included: limited knowledge about using technology and reservations about how and to what extent parents would use mHealth. Potential facilitators included nurses’ openness to learn and try new tools, confidence in their role and engagement in reaching parents as well as beliefs that the app could improve practice by prompting dialogue and being a shared platform.
24	van der Heiden et al., 2022 [36]	Primary care (The Netherlands)	Combined Lifestyle Intervention in primary care.	Not reported	15	GPs	6 (40.0%)	Not reported	Semi-structured interviews	Perceived facilitators were coordination of care provision by GP cooperatives and monitoring of the CLI implementation and their results. Reimbursement of CLIs without any costs for participants enabled application. Perceived barriers was the potential lack of added value of CLIs on top of existing lifestyle support.
25	Ware et al., 2012 [37]	Primary care (UK)	Positive Online Weight Reduction (POWeR): a web-based weight management programme. It is free to use and delivers tools and information to the patient online, minimising the need for practice staff training	Staff with experience with weight loss service delivery	36	Primary care staff (practice nurses *n* = 19; physicians *n* = 12; administrator *n* = 1; healthcare assistants *n* = 2)	24 (61.1%)	Not reported	Focus group interviews	Primary care staff felt under resourced to deliver weight reduction services to patients and unsure as to the effectiveness of their input, as routine services were not evaluated. Beliefs that current services were ineffective resulted in staff reluctance to allocate more resources.

Abbreviations: *TDR*, Total Dietary Replacement; *EHR*, Electronic Health Record; *PCP*, Primary Care Provider; *GPs*, General Practitioner; *LCD*, Low Calorie Diet; *T2D*, Type 2 Diabetes; *DiRECT*, Diabetes Remission Clinical Trial; *HCP*, Health Care Professional; *MCII*, Mental Contrasting and Implementation Intentions; *BMI*, body mass index; *PN*, Practice Nurse; *EWMM*, Eat Well Move More; *IBT*, Intense Behavioural Therapy; *PWMI*, Pediatric Weight Management Intervention; *HWC*, Health Weight Clinic; *M-HWYC*, Modified Healthy weight and Your Child

In total 572 HCPs were interviewed of which 42.7% were GPs, 29.4% practice nurses, 2.5% practice assistants, 7.9% dieticians and 0.52% physiotherapists [6, 21, 22]. Other studies also included health care directors and program managers [23], locality leaders [24] and health coaches (17.0%) [25] next to HCPs only. In most studies the participants were women [6, 21, 24, 26–37], though many studies did not report the sex of the participants [22, 23, 38–41].

Twenty of the included studies collected data by semi-structured individual interviews which were performed face-to-face or via telephone [21–25, 27–31, 34–36, 38–43], five studies conducted focus-group interviews [6, 26, 33, 37, 44], and one study combined questionnaire outcomes with interviews [32].

The interventions included weight management programs [21, 23, 32–34] including dietary and/or physical activity changes [6, 22, 24, 25, 28], addressing psychological health [31, 40], eHealth interventions including online guidance by HCPs [27, 41–43], and newly developed combined lifestyle interventions [36, 38, 39, 44]. Some interventions required a referral by GPs [26, 29, 30, 35, 37].

Most of the interventions focused on adults with overweight or obesity [6, 21, 22, 24–28, 31–33, 35–37, 40, 43, 44], six interventions were meant for children and their family [23, 30, 34, 38, 39, 41] and one focused on adolescents [29].

### Quality Assessment

Total scores ranged from 6 to 10 with an average of 8.2 ± 1.0 (see Table 2). Most studies did not fulfil the criteria to report the relationship between the researcher and the participants (*n* = 17), did not clearly explain their recruitment strategy (*n* = 11) or insufficiently explained the data analysis (*n* = 10).

**Table 2:** Scores from the quality assessment procedure using the CASP checklist

	1. Clear statement of the aims	2. Appropriate qualitative methodology	3. Appropriate research design	4. Appropriate recruitment strategy	5. Data collection	6. Relationship researcher and participants	7. Ethical issues	8. Data analysis	9. Clear statement of findings	10. Valuable research	Overall assessment
Alsaeed et al., 2022 [6]	1	1	1	0	1	0	1	1	1	0	**7**
Ayyaswami et al., 2024 [42]	1	1	1	0	1	0	1	0	1	1	**7**
Bennett et al., 2014 [26]	1	1	1	1	1	0	1	0	1	1	**8**
Blane et al., 2017 [21]	1	1	1	0	1	0	1	1	1	1	**8**
Brandt et al., 2018 [27]	1	1	1	0	1	0	1	1	1	1	**8**
Burton et al., 2023 [24]	1	1	1	1	1	0	1	1	1	1	**9**
Chimoriya et al., 2023 [28]	1	1	1	0	1	1	1	0	1	1	**8**
Cupit et al., 2021 [25]	1	1	1	0	1	1	1	0	0	0	**6**
Darling et al., 2023 [29]	1	1	1	1	1	1	1	0	1	1	**9**
Drew et al., 2024 [30]	1	1	1	1	1	0	1	0	1	1	**8**
Finn et al., 2024 [38]	1	1	1	0	1	1	1	0	1	1	**8**
Govindasamy et al., 2023 [31]	1	1	1	1	1	0	1	1	1	1	**9**
Johnson et al., 2018 [39]	1	1	0	0	0	1	1	0	1	1	**6**
Nederveld et al., 2020 [40]	1	1	1	0	1	0	1	1	1	1	**8**
Paine et al., 2023 [44]	1	1	1	1	1	0	1	1	1	1	**9**
Parker et al., 2024 [32]	1	0	0	1	1	0	1	1	1	1	**7**
Persaud et al., 2022 [23]	1	1	1	1	1	0	1	1	1	1	**9**
Poppe et al., 2018 [43]	1	1	1	1	1	0	1	1	1	1	**9**
Porter et al., 2021 [33]	1	1	1	1	1	0	1	1	1	1	**9**
Rehackova et al., 2022 [22]	1	1	1	1	1	1	1	0	1	1	**9**
Simione et al., 2020 [34]	1	1	1	1	1	0	1	1	1	1	**9**
Smith et al., 2017 [35]	1	1	1	1	1	1	1	0	1	1	**9**
Thomas et al., 2022 [41]	1	1	1	1	1	1	1	1	1	1	**10**
Van der Heijden et al., 2022 [36]	1	1	1	0	1	0	1	1	1	1	**8**
Ware et al., 2012 [37]	1	1	1	0	1	0	1	1	1	0	**7**

### Qualitative Findings

After thematic synthesis, 382 codes were identified and assigned to 323 marked quotations. These codes were grouped into seven themes, divided between barriers and facilitators. The seven main themes identified were logistical challenges to implement the intervention, intervention characteristics, support, patient-related factors, provider-related factors, financial resources and effectiveness of the intervention. The themes and codes with corresponding quotes are presented in Table 3.

**Table 3:** Overview of themes and codes with corresponding quotes

Thema	Code	Examples of quotes
Logistical challenges [22, 24, 26, 30–33, 35, 38, 41–44]	Lack of time and extra work	*“I think the only thing I’ve found is it’s quite time-consuming. So*,* although we know how beneficial it can be*,* and we really want to promote the programme and to get patients onto the programme*,* it is quite time consuming…”* [30] *“It was a time commitment…with my work schedule*,* it was just a little bit too much… Time*,* for me*,* was a barrier.”* [38] *“Well*,* for those of us who work full schedules*,* and let’s say we’re seeing 25 patients a day. It’s prohibitive for me to have another something that I have to clock into to pull up information to check into. My practice module hardly permits me enough time to eat and sleep*,* so I wouldn’t want another thing to do for patient to come in*,* going into another system*,* pulling up something out of a – but it would be good to know this [information about patients’ weight management] if I had time set aside for this.* [26]
[22, 24, 26, 30–33, 35, 38, 41–44]	Extra work	*“I can see the utility in this. Somebody’s keeping an eye on it*,* they may be more likely to follow through with those recommendations*,* if they buy in. I can see it being a quick thing that you could send a note to the patient and either encourage them or congratulate ‘em on how they’re doing. The concern with it is that the inbox is already cluttered. Depending on the frequency of it*,* it becomes one more thing that you need to deal with.”* [42] *“[At] the time the study was introduced to us*,* we were about to start a diabetic clinic…it got so overwhelming…the doctors dropped from that study…when this study was introduced*,* the practice was reluctant*,* but we [nurses] wanted to*,* as we thought it will be beneficial…I explained it to the practice manager and they said ok. But is was overwhelming at times because of the paperwork.”* [31]
[21, 24, 30, 32, 33, 36, 38, 40, 43]	Lack of staff availability	*“We don’t have the extra manpower sitting around to do it.”* [33] *“I think we have to look at things that have value and things that we can do. We’re doing a lot of things. So*,* that’s why there’s some hesitation here. Do we have the capacity to do that?* [33] *“When we set it up there was a lot of people around the table saying ‘we don’t want to promote this heavily because we think we are going to be inundated’.”* [21] *“Certain surgeries have their own problems with staffing or engagement*,* particularly with diabetes and things like that. But it is just a national issue when it comes to primary care.”* [24] *“I don’t have a behaviourist. I have a social worker that I work with*,* but this is not her wheelhouse at all. Yeah*,* so that is not an option. I would just say access*,* I didn’t have anybody that I could use.* [38]
[31–33, 43]	Lack of facilities	*“That was the only bad thing*,* I didn’t have one set room…when I am asking questions and I get called over to assist in something else. That was the hardest thing. I was expected to do my regular work around it*,* you have doctors coming in and saying that*,* and you say excuse me*,*…”* [31]
Intervention characteristics [23, 24, 26–28, 30, 32, 34, 35, 39]	Poor communication between HCPs and patients and/or program leaders	*“A couple of [my patients] wanted to talk about it a lot*,* so to them*,* I guess*,* it was important that I knew all the details. But*,* since there wasn’t any direct contact between us and program [staff]*,* like no emails*,* no – when we send someone out for a consult we get a letter back*,* so I feel like a little more continuity – I didn’t have that. So it felt like*,* put them out into the study and now they’re your baby. So yeah*,* [I was] not so involved.”* [26] *“All I got to see was their goals and any weights they’ve logged in […] if they’d made any comments about how they’d had a particularly good week or bad week or anything like that*,* might’ve been quite interesting*,* just to have sort of been able to see that as well.”* [35]
[23, 24, 26, 28, 32]	Lack of engagement general practice	*“We have briefly discussed about how to encourage other GPs and other healthcare professionals…If we could get the GPs together*,* get them to sit down with the endocrinologist and the dietitian and tell them what the programme is about and what we are going to expect once we get the patients enrolled and…who do we contact if you have feel like you know a lot more before you offer the programme to your patients.”* [28]
[6, 24, 30, 34, 35, 38, 41, 43]	Lack of intervention tools	*“I personally think that it would be really nice to have something that we can hand them at the visit*,* some educational materials and information about local resources…I think all of it is going to be beneficial.”* [34] *“And especially if there is something we use these pictures…or if you have difficulties with the language or…then it is very good to have a picture.”* [41] *“Because most of them are obese*,* so patients with obesity perceive solid foods as essential. I mean if it was food*,* it would be better*,* but only liquids? That’s difficult.”* [6] *“It’s just a liquid diet really which doesn’t sound appealing*,* even if you’re engaged. It looked pretty good to me*,* and most people are aware of meal replacement kind of regimes*,* for better or for worse.”* [24]
[24, 30, 36]	Complex referral process	*“It has been a challenge*,* getting referrals in*,* you know*,* a referral takes at least 20 minutes for a GP.”* [24] *“Half of referrers*,* mainly practice nurses and pharmacists*,* discussed generally having limited knowledge of how those referred were progressing on the programme*,* which both led to some uncertainty about the care needs of individual patients as well as about the programme more broadly.”* [30] *“If you’d spoken to me at the beginning my answer would have been very different. I was quite frustrated initially because its seemed like every referral I sent got back to me. And obviously it took time to do all the referrals and we had a list of patients that wanted to be referred.”* [30]
[6, 26, 28]	Length of the intervention	*“For [my patient] it was the intensity and frequency of the feedback…We’ll see people 3 to 6 months [in clinic]*,* and there’s just too much of a lag time in between when you’re giving advice and getting feedback…We can’t see people every month.”* [26] *“Quite a lot of patients do meal replacement by themselves*,* they get stuff from the market*,* but they’re not exactly sure how to do it. Doing this together with the clinicians involved*,* the dietitians involved*,* I think it was quite motivating for them. Definitely I can see the benefit of longer-term support for them to help maintain that weight and remission*,* keep the medication off and I would like to see that it’s effective.”* [28]
[35, 37, 42]	Electronic communication	*“I suppose the face-to-face is nicer from the relationship you get with the patients*,* but on the other hand the email can be perfectly efficient and a lot of people lead very busy lives*,* they don’t want to be coming in.”* [35] *“Having that order set is great…what I fear about [getting notification in] my Chart messages is that it is just more work to do. I like the fact that you’re able to identify how often you get the alerts because I can just time it for another visit in one month.”* [42]
[24, 27, 32, 35, 39]	Lack of feedback	*“It would be nice to get more collated feedback from [service provider] about the people we’ve referred because it’s been sporadic.”* [24] *“The only thing that I find sometimes a little bit frustrating is that you don’t have any feedback as to how well the programme went; did the family or child lose weight or didn’t they attend; that’s the only criticism I would have for the programme.”* [39]
[6, 21, 26–28, 34–38, 41, 43]	Clear communication between HCPs and patients; and HCPs and intervention program	*“We met with [intervention] for almost a year…it was coordination of all the processes involved including the curriculum. And that was one of the most valuable processes for us*,* tor the administration. It meant that the program was connected to the [intervention program]*,* we got the feedback from those folks who were wonderful listeners to us.”* [38] *“I think that I was the factor that made the difference*,* since he [the patient] knew that I was the person who was coaching him. He had met me in person and it made a difference that it was not just another app he could use for entering his data. Here*,* he actually got concrete answers to his questions.”* [27]
[21, 26, 27, 34, 35, 37, 41, 43]	Combination of electronic and face-to-face contact	*“I suppose the face-to-face is nicer from the relationship you get with the patients*,* but on the other hand the email can be perfectly efficient and a lot of people lead very busy lives*,* they don’t want to be coming in.”* [35] *“I still I think a lot of it is down to the communication aspect against and so I think that doing more face to face communication with people and raising awareness*,* so whether it’s*,* you know*,* attending whatever kind of meeting so that you can have more of a conversation about it would be helpful from that point of view because I think*,* I do think*,* you know*,* email*,* etc. has its place and it is very useful but I don’t think anything*,* you know*,* kind of compared to face to face.”* [21]
[6, 26, 27, 34, 37, 38, 43]	Insight in patients’ progress	*“I think my patient liken when I showed [him] the progress*,* the charts at me as I’m their cheerleader. […] During the visits we recognized what they were doing*,* encouraged them to stick with it*,* and a number of them did well.”* [26] *“…If you can access the patient details on there*,* you can see how they are progressing*,* then I guess everyone is singing from the same hymn sheet aren’t they?”* [37] *“I think for me what was different about the trial was that there was a…sense of coherence between [us] – it’s the same institution. We recruited the patients*,* identified the patients*,* so…their joining became a larger part of their overall treatment plan. When I’ve had patient go into research trials before*,* it’s been sort of like a black box*,* so you may get reports back*,* but you really have a fuzzy sense of what happens and what their commitment is in that trial*,* and that wasn’t the case. It was more like a glass box in the sense that there was a back-and-forth communication and people actively talked about their progress in a way that I could understand it*,* so I thought it was different than the typical referral of a patient to a blinded trial.”* [26]
[21, 26, 27, 29, 30, 44]	Referral by primary care to program (easy referral system, clarity about referral)	*“We’ve got a very useful template which maybe articulates what you need to do for the referral so that’s very useful. It’s easily emailed using our AccuRx which is useful […] so that means that I can email straight away during the consultation rather than having to ask somebody else to email which happens for some outside organizations. So*,* I would say the useful template*,* a fairly quick and easy form*,* makes things easy.”* [30] *“What’s interesting is that where there has been long term sort of work between the local authorities and the GPs and practice nurses in the area they are getting much better referrals coming through. So where there is already a partnership*,* a relationship built up*,* they are getting*,* you know*,* they are getting frequent referrals coming through. In the areas where that’s not as well established then you can kind of see the difference.”* [21] *“I think the referral was powerful*,* and I think it was nice for patients to be able to say this one is recommended by my doc*,* so there’s got to be some validity to this.”* [26]
[23, 24, 28, 38, 43]	Multi-disciplinair team	*“They’ve [participants] got the support of myself [practice nurse] and the dietician. Couple of them contact the dietician*,* and they contact me and just say*,* look this is the situation*,* and then we can give them that encouragement. We are there for them*,* and they’re not doing it alone.* [28] *“I suggest we the pharmacists on board because the pharmacists probably had a bit more time available…and usually that worked well.”* [24]
[6, 23, 26, 29, 39, 41]	Patient/family centered program	*“I explain to them that the weight management program encompasses the whole person treatment of obesity. Not only physically and dietary wise*,* but also emotionally.”* [29] *“But again it’s hard because that child is not…or young person is not incontrol of the cooking at home. And I’m guessing that along with overweight children you’ve probably got overweight parents*,* so it’s not just about impacting that one child it’s about trying to get the whole family on board.”* [39] *“So I think that there needs to be more focus on the parents and educating them because they’re coming from a family*,* you know*,* they’re in the same situation. So some parenting skill*,* limit setting*,* cooking*,* shopping*,* and menu planning.”* [23]
Support [25, 26, 30, 32, 35, 40, 41]	Provided trainings	*“I wasn’t on the initial training about the programme*,* a handful of the GPs were. But one of the partner GPs*,* she’s the diabetes kind of lead*,* she’s the one I discuss mostly with and she told me about it first.”* [30] *“What is needed it that everybody works with the app the same way…because sometimes we meet each other’s children and…so it is important that everybody has the same training so we work the same way…refer similar cases.”* [41] *“My GP partner and I have found that this is a completely and utterly joyful way of doing medicine. We used to joke about our exit plan. We now tell people how liberating it is to practice medicine like this. You’ve got people coming into your room saying ‘I can’t lose weight’*,* ‘that’s a heart-sink’*,* ‘I’ve got chronic pain*,* irritable bowel*,* I’ve reflux*,* diabetes I can’t control’. Now all of a sudden we have this magnificent tool in the box. It’s completely changed our lives as practitioners.”* [25]
[35, 43]	Reminders	*“It’s useful to have the emails just to flag up and I used to leave them in my inbox just as a reminder until the patient you know had been dealt with.”* [35]
[23, 32, 34, 39, 43]	Availability of intervention resources	*“I found this a very interesting project. Especially because the researchers did all the preparatory work and gave us a ‘ready-to-use-package’ and programme that provided all the tailored feedback. I really liked it.”* [43] *“Not just pointing pictures at the book*,* but having the physical food there and the key was*,* portion control*,* so seeing what a plate looked like.”* [23] *“I am interested in the text messaging program*,* I feel like [parents] communicate that way the most. I don’t think an email would be effective…handouts are easy for us.”* [34] *“A visual prompt in the GP office such as a chart or characterisations of body shapes.”* [39]
Patient related factors [6, 21, 28–31, 38, 39, 43, 44]	Lack of motivation and engagement patients	*“We may have a role to convince people to try it [the intervention]*,* but it al depends on the individual’s motivation*,* is it sustainable? He may commit one week and then five up.”* [6] *“I know that the weight management program is rather rigorous and it has certain expectations of teens and families…I might not emphasize it as that great of an idea for [somebody that didn’t seem motivated].”* [29] *“They just don’t want to stop eating food altogether you know for a that time because they might have weddings*,* I had one person say well I’ve got a wedding in a couple of weeks*,* and I’ve got a christening here and I’m going to a party there and I want to eat a bit of cake and things like that you know. So*,* I think that stops it a little bit*,* people don’t want to stop eating for that amount of time […] they don’t want to live on shakes and soups.’* [30] *“But you’re falling that child in a way aren’t you. And the referral to Eat Well is stopped because there’s no parental consent*,* so you can’t…we see them in drops ins and things and healthy plate and healthy eating and give hem the leaflets*,* but…”* [39] *“I fins the ones that have the overweight issues*,* the story’s always the same: ‘I don’t want to move’*,* ‘It hurts’; and I realise I’m overweight but*,* um*,* it can’t hurt this bad*,* and thinks like that. All the excuses.”* [44]
[23, 24, 26, 29, 33, 35, 37, 38, 41–44]	Logistical challenges for patients	*“The couple first questions I always get are: where is it located*,* does my insurance cover it*,* what does it look like*,* am I going to be sitting in a classroom.”* [29] *“People definitely ask*,* who has the time and money to make some of these things happen.”* [29] *“I guess you have got to try to work in with general patients*,* try and talk to them at their level. Sometimes it’s hard to work out*,* you know*,* what someone’s level of literacy it.”* [44] *“I selected my patients based on whether I knew they could work with a computer. I could for example not ask persons of 80 years or older*,* for whom I know cannot work with or do not have a compute*,* to participate in this study. However*,* I did not use any other selection criteria.* [43] *“If they’re coming from the shore or 45 minutes away…there is just no way for them to come here and doo the program because it is intensive. There is no other program in [state] that would be able to do what our weight management program does.”* [29]
[6, 23, 24, 29, 31, 34, 38, 41–44]	Patient characteristics	*“I think it depends on the age group*,* I mean especially educated people who are well-read and are ready to get rid of their diabetes*,* yes. But elderly patients are used to their daily routine and medications and whatever*,* do not go and tell them to change.”* [6] *“We have asked if the materials for the course are suitable for people with learning disabilities…and what they have said it they don’t have any videos. People can come along and join the programme*,* however*,* there is a level needed as to whether they retain*,* understand all of the information.”* [24]
[6, 21, 28, 31, 44]	Attitude of patients	*“They [patients] were like you know that’s best for me*,* you know the right thing to do […] just tell me how I can fix my diet or my way of eating.”* [31] *“I think they look up to doctors and nurses…who give them instructions and [patients] just follow it. They lived like that their whole life. There is no discussion*,* doctors do not discuss with them […]. They expect you to give them direction*,* so that they can just follow it.”* [31]
Provider related factors [22, 29, 32, 37, 40–42, 44]	Lack of knowledge and training	*“I haven’t really had too much like training in it specifically*,* but I have a strong interest in exercise and fitness and everything in general. I suggest starting really low with basic type of things. But when it comes to patients that have trouble walking or doing exercises because of pain*,* I have a really hard time with helping them because I don’t know what to suggest.”* [42] *“I don’t have the training to give them what they really need – I mean I can start the conversation*,* sure*,* and I’ve had plenty of patients take what I’ve told them and run with it*,* and do really well for themselves*,* but most people they need a health coach*,* they need a dietician.”* [40] *“…I don’t think I have had any formal weight loss reining*,* I just go on what I do myself*,* and just things that you have read and […] common sense.”* [37]
[21, 39]	Lack of awareness	*“I think it probably could do with a bit of a re-launch with the general practice population. Because it’s service you don’t use very often you tend to sort of forget it exists.”* [39] *“I know it’s a referral system which I think patients can refer themselves*,* if I remember rightly*,* and it’s kind of to do with eating and exercise and management of weight problems*,* yes?…I think I was probably thinking about something slightly different from Eat Well because this was a couple of years ago…”* [39]
[6, 28, 29, 32, 41]	Lack of confidence	*“I don’t think I’m brave enough to give them [TDR]*,* because it’s 800 calories a day! I won’t be able to do it”* [6] *“I have gained so much from them [bridge builders]…a lot…a culture competency which I didn’t know…yeah didn’t realise existed before I started here.”* [41]
[26]	Lack of feeling involvement	*“Though I felt…fairly remote from the system [of the intervention program]*,* except that I had a sense of relief that these patients were engaging in a process that I thought might potentially be useful. So it made me feel good to know that they were doing something about it-in each of these cases*,* there were some really significant comorbidities that they were doing something about it which they weren’t otherwise doing. But what sort of positive role I had for them*,* I don’t really know”.* [26] *“She did great. And she did it with the help of the coach*,* and not from any input from me.”* [26]
Financial resources [26, 33, 36, 40, 42]	Lack of insurance coverage	*“I’d wanna be sure that I wasn’t prescribing something to patients that would cause them a lot of financial burden. I’m not even sure if this is covered by insurance. Is this something that I would be stick my patients with a $200 device when they only make a few hundred bucks a week?”* [42] *“I believe the most frustrating part of this is some insurers*,* they pay for the visit if the BMI’s over 30*,* but now we do a good job and the BMI’s under 30*,* now they won’t pay for the visit anymore because they’re not obese. If we were managing hypertension*,* and then we got their blood pressure under control*,* we could still bill for hypertension.”* [40]
[21, 23, 26, 33, 36, 38, 40]	Lack of funding and reimbursement	*“It [CLI] will not get off the ground*,* because they have deliberately limited budget.”* [36] *“We know for a fact that we will not have any physio input without funding*,* we won’t have any psychological input without funding and even simple thinks like venues and resources we are fairly limited for that as well.”* [21] *“There was always a struggle with the financial support and funding.”* [38] *“I think it’s very important that health insurance be providing reimbursement…it would create a priority*,* for different organizations to provide these services. If they can’t find the funding*,* they won’t be able to put more into it.”* [23] *“The big factor going forward is going to be how people get reimbursed because obviously [clinic staff] can’t be taking out large chunks of their day and not get paid for it.”* [33]
[36]	Long-term financial support	*“I hope that when health insurance companies say we will reimburse it*,* they will do so for at least 5 years or so. That there is the opportunity to build something and have success with it. Because I think*,* it takes around 2–3 years before such a new measure is picked up a bit.”* [36]
Effectiveness of the intervention [23, 24, 28, 30, 36, 40]	Lack of evidence-based outcomes	*“I think it is important*,* and making sure it’s evidence-based*,* which I think goes part and parcel with the cost…efficiency and quality equation*,* but then*,* going that step further to say*,* ‘Let’s not look at it as a one-year*,* how much did you save*,* but in the long run.”* [23] *“I thought the DIRECT trial was astonishing…but we can’t 100% hand on heart say we know that it’s going to actually save your life because we haven’t got the data yet.”* [24] *“I think we just have to recognize this is a lifelong*,* relapsing*,* chronic disease. There have to be treatments available through a lifetime*,* and when you look at the data of long-term weight maintenance it’s really poor…this is complicated*,* and it’s multi-factorial*,* and it’s difficult*,* and it isn’t about what you eat.”* [40] *“When I refer my patients to Desmond […]*,* I know exactly what they’re going to experience on that day or the two day programme because I went along as a health care professional to see what they’re being taught. Whereas with the low calorie diet [intervention]*,* I don’t have that information.”* [30]
[22, 25]	Proven effectiveness of the intervention	*“It was a great opportunity professionally for the members of the team to develop and*,* I suppose reputationally for us to be involved in a trial that was hopefully going to be very effective.”* [22] *“When I heard his [principal investigator’s] presentation and the idea of what he’ discovered with his first trial*,* the Newcastle Study*,* and he talked about that and the changes he’d noticed within the pancreas and the liver in that*,* that I must admit was the seeing his picture*,* images of that change in*,* the rapid change*,* in the fatty infiltration of the pancreas*,* that absolutely persuaded me.”* [22]
*GPs*, General Practitioner; *TDR*, Total Dietary Replacement; *CLI*, Combined Lifestyle Intervention; *DIRECT*, Diabetes Remission Clinical Trial.		

#### Logistical Challenges

Logistical challenges were mentioned as barrier for the implementation of lifestyle interventions in primary care by 16 studies [21, 22, 24, 26, 30–33, 35, 36, 38, 41–44]. The main challenges reported by HCPs were lack of time to provide the intervention, additional workload for HCPs and lack of staff available to provide the intervention. Lack of facilities such as no available room in practice and poor or no internet connection to provide online guidance were reported as barrier mainly by GPs and practice nurses.

#### Intervention Characteristics

Characteristics of the intervention were mentioned by 23 studies, of which 17 studies reported certain intervention characteristics as barriers [6, 23, 24, 26–28, 30, 32, 34–39, 41–43] and 18 reported facilitators [6, 21, 23, 24, 26–30, 34–39, 41, 43, 44]. The main barriers reported by all types of HCPs were poor communication between HCPs and patients or with the intervention program after referral, lack of engagement of HCPs, poor intervention tools which were not evidence-based nor up-to-date or available in multiple languages, complex referral processes, length of the intervention, electronic communication and lack of feedback. The most reported facilitators were clear communication towards patients and between HCPs, combination of electronic and face-to-face contact, insight in patients’ progress, referral by GP to the intervention program via an easy referral system and a multidisciplinary team. A patient/family-centred program was mainly reported as facilitator for interventions targeting children and adolescents.

#### Support

In thirteen studies, the support provided to HCPs worked as facilitator to implement and to provide the lifestyle intervention in primary care [6, 22, 23, 25, 26, 30, 32, 34, 35, 39–41, 43]. Specifically, the training offered to HCPs enhanced their knowledge and skills on the subject which improved their confidence to provide lifestyle interventions to patients. The availability of resources such as information materials, intervention curriculums, example health advices supported HCPs. Reminders which were sent about the interventions to especially GPs, improved their awareness and alertness to refer patients to or to provide the lifestyle intervention.

#### Patient-related Factors

Nineteen studies reported patient-related factors which were perceived as barriers by all types of HCPs to implement the intervention [6, 21, 23, 24, 26, 28–31, 33–35, 37–39, 41–44]. The main patient-related reported barriers were lack of patient motivation and engagement of patients, attitude of patients, logistical challenges for patients like costs, transport, technology access, patients’ characteristics such as their age, educational level or comorbidities.

#### Provider-related Factors

Provider-specific related factors which made it difficult to implement the intervention in practice were reported by 12 studies [6, 21, 22, 28, 29, 32, 37, 39–42]. The following factors were mainly reported by GPs, practice nurses, dieticians and paediatricians: lack of knowledge, lack of awareness and lack of confidence to provide lifestyle intervention. Also the lack of feeling of involvement was reported as barrier by GPs and practice nurses.

#### Financial Resources

Eight studies reported the lack of financial resources as barriers for the implementation of a lifestyle intervention in primary care [21, 23, 26, 33, 36, 38, 40, 42]. The lack of insurance coverage for patients and the lack of funding or reimbursement for primary care providers were reported as main barriers for GPs, practice nurses and dieticians to implement lifestyle interventions in primary care. Whereas when financial support was provided to primary care, it was reported as facilitator by GPs to implement the lifestyle intervention in practice [36].

#### Effectiveness of the Intervention

The lack of effectiveness of the intervention was reported as a barrier in six studies [23, 24, 28, 30, 36, 40]. The unavailability of evidence-based outcomes on the long-term effectiveness of the lifestyle intervention made it difficult for HCPs to inform, refer and to provide the intervention to eligible patients in their practice. While the availability of long-term effects of the lifestyle intervention was perceived as a facilitator by HCPs [22, 25]. Proven effectiveness of the intervention stimulated and motivated HCPs to implement and to provide the intervention in practice to their patients.

## Discussion

### Main Findings

This systematic review of qualitative studies investigated the barriers and facilitators of HCPs with implementing lifestyle interventions for patients with overweight or obesity targeting weight loss in primary care. The thematic synthesis of the 25 included studies identified seven key themes: logistical challenges, intervention characteristics, support, patient- and provider-related factors, financial resources and effectiveness of the intervention. The reported barriers and facilitators were consistent across the different types of HCPs and were similar for interventions focusing on children, adolescents, and adults.

### Interpretation of the Findings

Organizational challenges are often reported as barriers for the implementation of lifestyle interventions in primary care [45, 46]. In this review, logistical challenges such as lack of time, shortage of staff, or poor information and communication technology (ICT) facilities hampered the implementation of lifestyle interventions into practice. HCPs expressed that due to these challenges the intervention often did not align with their workflow. Workflow refers to the processes, settings and tasks HCPs follow to provide patient care and manage administrative duties. It has been demonstrated that when ICT is supported to integrate the intervention with practices’ electronic medical system, along with the flexibility for HCPs to adjust time slots and patient appointment durations, successful implementation is facilitated [46, 47]. To achieve a ‘good fit’, future interventions should therefore consider the workflow of the involved HCPs to positively influence the implementation.

Good communication between HCPs and patients is essential for successful implementation of a lifestyle intervention in primary care [6, 21, 26–28, 34–38, 41, 43]. To enhance communication between HCPs and patients, electronic communication such as online platforms and email contact is upcoming [48]. However, in this review mixed experiences in utilizing online platforms to provide the intervention were observed. Several HCPs mentioned that electronic communication feels less personal, responding to chat messages involves considerable extra work, and feedback from colleagues was often lacking. A study on the acceptability of an online weight management program in primary care showed that online platforms could encourage patients to actively take part in the intervention, but that many HCPs will not have the time to use it and insights in patient’s progress is often missing [49]. To improve communication to enhance provision and implementation of the intervention, it is recommended to combine electronic and face-to-face interactions, to allow insight in the patient’s progress and to schedule regular contact moments in order to provide feedback between providers.

In this review, many studies reported that HCPs, especially GPs, practice nurses and dieticians, struggled to provide lifestyle interventions to their patients due to a lack of knowledge and skills. It has been shown that HCPs often lack knowledge in lifestyle and exercise recommendations or are unfamiliar with techniques to facilitate behavioral changes in patients [50–53]. A review on experiences of HCPs with weight management reported that HCPs were unaware about dietary advices or did not receive essential training needed to provide weight management [54]. Interestingly, the few studies included in this review that provided training or educational materials to HCPs observed improvements in HCPs’ skills and confidence. Thus, trainings for HCPs should be offered with the intervention since these are essential to improve their knowledge and skills to confidently provide the intervention and improve implementation. Next to trainings, the provision of clear and easy to use tools could assist HCPs [55, 56]. Though, this review showed that many interventions lacked supportive tools or information resources for HCPs. HCPs complained that resources were often not up-to-date, nor applicable to their patients or were not provided. To equip HCPs with tools such as intervention handbooks enhanced their perceived self-efficacy in providing weight management care [57]. Therefore, it is strongly recommended that, in addition to training, clear and appropriate tools will be provided to HCPs to support them providing and implementing the intervention.

An important factor mandatory for successful implementation of lifestyle interventions in primary care are financial resources. However, this review identified that financial support for primary care to implement lifestyle interventions is often limited [58–60]. This lack of financial reimbursement impeded HCPs to get the intervention off the ground or to deploy extra staff to provide the intervention. Consequently, multiple HCPs were not motivated to provide the intervention since there was only a minimal financial compensation for all the hard and extra work. Furthermore, this review showed that unclarities about the coverage of the intervention by the insurance of their patients deters HCPs to inform them about the intervention. Whereas clear information about insurance coverage is essential as insurance coverage can reduce the barrier for HCPs to refer patients to lifestyle interventions [61, 62].

### Strengths and Limitations

To the best of our knowledge, this is the first qualitative systematic review that identified the barriers and facilitators of HCPs to implement a lifestyle intervention for patients with overweight or obesity targeting weight loss in primary care. The results of this review are likely generalizable to a broad population since the lifestyle interventions were conducted in various countries including diverse study populations. However, these findings are primarily more applicable to western countries with comparable health care systems and should therefore be interpreted with caution. For this review a rigorous search strategy was used by searching four large databases and mainly high quality studies were included.

There are some limitations that should be taken into consideration. It is important to interpret the results of this review with caution since the included studies differed in delivery mode (eHealth) and intervention study population (adult vs. child and family), and not all studies clearly specified the type of included HCPs. Furthermore, eligible studies could be missed since references of included studies were not checked.

### Clinical Implications

Up-to-date tools such as handbooks, information resources or illustrations which are available in multiple languages, considering differences in education levels and literacy of patients should be provided to HCPs. Although eHealth interventions are upcoming, it is essential to enable a combination of electronic- and face-to-face contact between HCPs and patients to enhance communication and improve implementation. Sustained financial support could positively impact implementation in primary care. To persuade governments, municipalities, and health insurance companies to allocate more financial resources, further studies are needed to demonstrate positive long-term health outcomes of lifestyle interventions which will reduce healthcare costs and decrease the high pressure on the healthcare system.

### Future Research

Before further research is conducted to investigate the feasibility and applicability of lifestyle interventions in primary care, policy makers should address the barriers and facilitators identified in this review otherwise they will likely be faced again. Policy makers should ensure that infrastructure, logistical, and financial resources are in place before further research will be proceeded. This is a significant challenge, particularly because primary care systems vary by country. Nonetheless, addressing these barriers is essential for the successful implementation and continued research of lifestyle interventions in primary care.

## Conclusion

This systematic review identified the main barriers and facilitators for HCPs to implement a lifestyle intervention for patients with overweight or obesity targeting weight loss in primary care. Interventions that fit within the HCPs workflow is fundamental for successful implementation of the intervention by offering structural support, training and supportive tools to HCPs. Furthermore, the possibility to communicate electronic or face-to-face and sustained financial support enhance implementation of the intervention in primary care. Policymakers should ensure that the necessary conditions are in place before future research on implementing lifestyle intervention in primary care will be conducted.

## Key References

- Oosterhoff, M., de Weerdt, A. C., de Vries, E., Feenstra, T. & de Wit, A. Annual report on the combined lifestyle intervention (CLI) monitor 2024. National Institute for Public Health and the Environment, doi: https://rivm.openrepository.com/handle/10029/627991 (2025).
  - ○ Lifestyle interventions showed to be effective treatments to help patients with overweight and obesity to achieve a healthier bodyweight and to improve their quality of life, however, active participation of patients and the involvement of staff are crucial.
- Wharton, S., Lau, D. C., Vallis, M., Sharma, A. M., Biertho, L., Campbell-Scherer, D., & Wicklum, S. Obesity in adults: a clinical practice guideline. Cmaj 192, E875–E891, doi: 10.1503/cmaj.191707 (2020).
  - ○ Primary HCPs serve as the initial point of contact for patients and frequently establish a long-term trustful relationships. HCPs therefore play a pivotal role in the prevention, management and treatment of overweight and obesity.
- de Jong, M., Jansen, N. & van Middelkoop, M. A systematic review of patient barriers and facilitators for implementing lifestyle interventions targeting weight loss in primary care. Obes Rev 24, e13571, doi: 10.1111/obr.13571 (2023).
  - ○ This review reported main barriers and facilitators experienced by individuals with overweight and obesity for implementing lifestyle interventions in primary care and patients emphasized the crucial role and involvement of primary care staff for successful implementation.
- Grady, A., Jackson, J. K., Lum, M., Delaney, T., Jones, J., Kerr, J. & Yoong, S. Barriers and facilitators to the implementation of healthy eating, physical activity and obesity prevention policies, practices or programs in family day care: a mixed method systematic review. Prev Med 157, 107011, doi: 10.1016/j.ypmed.2022.107011 (2022).
  - ○ Addressing barriers and facilitators such as resource availability, and social and environmental factors are essential for effective implementation of lifestyle interventions aimed at promoting healthy eating, increasing physical activity, and strengthening obesity prevention programs among patients with overweight and obesity.

## Supplementary Information

Below is the link to the electronic supplementary material.

Supplementary Material 1

## Data Availability

No datasets were generated or analysed during the current study.

## Abbreviations

HCPs: Health Care Providers
GPs: General Practitioners
WHO: World Health Organization
BMI: Body Mass Index
SD: Standard Deviation
CASP: Critical Appraisal Skills Program
ICT: Information and Communication Technology
