# Supplementary material for: Health Care Professionals’ Barriers and Facilitators for the Implementation of Lifestyle Interventions Targeting Weight Loss in Primary Care – A Systematic Review of Qualitative Studies
Source: Curr Obes Rep. 2026 Jun 4;15(1):43. doi: 10.1007/s13679-026-00721-8 (PMC13236802; doi:10.1007/s13679-026-00721-8)
Supplement: Supplementary file 1 — Supplementary Material 1 [file 13679_2026_721_MOESM1_ESM.docx]

**Appendix**

**Appendix S1: Search strategy**

**Embase.com**

('family medicine'/de OR 'general practitioner'/de OR 'general practice'/de OR 'primary health care'/exp OR ((family NEXT/1 (medicine OR doctor*)) OR ((general OR family) NEXT/1 (practi* OR physician*)) OR (primary NEXT/2 (healthcare OR health-care OR care OR medical-care)) OR gp OR gps):ab,ti) AND (lifestyle/exp OR 'lifestyle modification'/de OR 'healthy diet'/de OR exercise/de OR 'physical activity'/de OR 'motor activity'/de OR 'weight loss program'/de OR 'body weight loss'/exp OR 'health promotion'/de OR 'health program'/de OR 'fat intake'/de OR 'caloric intake'/de OR 'eating habit'/de OR 'body weight management'/de OR 'health behavior'/de OR 'food intake'/de OR (lifestyle* OR life-style* OR ((health* OR habit*) NEAR/3 (diet* OR eating)) OR exercise* OR sedentar* OR ((physical* OR motor) NEAR/3 (activ* OR inactiv*)) OR (weight NEAR/3 (loss OR losing OR control OR reduc* OR management*)) OR (Intervention* NEAR/3 program) OR (health* NEAR/3 (promot* OR program*)) OR ((fat OR calori* OR energ*) NEAR/3 intake) OR (behave* NEAR/3 chang*) OR ((fruit* OR vegetable*) NEAR/3 consum*)):Ab,ti) AND (obesity/exp OR 'body mass'/de OR 'body weight'/de OR 'weight loss program'/de OR 'waist circumference'/exp OR 'waist hip ratio'/exp OR 'body weight control'/de OR (obesity OR obese OR overweight OR BMI OR body-mass* OR (weight NEAR/3 (loss OR losing OR control OR reduc* OR management*)) OR (waist NEAR/3 (hip OR circumfer*))):ab,ti) AND ('implementation science'/de OR barrier/de OR facilitator/de OR 'program evaluation'/de OR 'feasibility study'/de OR 'pilot study'/de OR 'health personnel attitude'/exp OR 'patient attitude'/exp OR motivation/exp OR 'clinical audit'/de OR (implement* OR barrier* OR facilitator* OR (program* NEAR/6 evaluat*) OR feasib* OR pilot OR reluctan* OR attitude* OR motivat* OR refus* OR adher* OR nonadher* OR complian* OR noncomplian* OR (patient* NEAR/6 (characteristic* OR view* OR want OR perception*)) OR willingness* OR clinical-audit*):Ab,ti OR evaluat*:ti) NOT ([conference abstract]/lim AND [2000-2018]/py) NOT ([animals]/lim NOT [humans]/lim)

**Medline ALL Ovid**

(General Practitioners / OR exp General Practice / OR Primary Health Care / OR ((family ADJ (medicine OR doctor*)) OR ((general OR family) ADJ (practi* OR physician*)) OR (primary ADJ2 (healthcare OR health-care OR care OR medical-care)) OR gp OR gps).ab,ti.) AND (exp Life Style / OR Diet, Healthy / OR Exercise/ OR Motor Activity / OR Weight Reduction Programs / OR Weight Loss / OR Health Promotion/ OR Diet, High-Fat / OR Diet, Fat-Restricted / OR Energy Intake / OR Feeding Behavior / OR Health Behavior / OR (lifestyle* OR life-style* OR ((health* OR habit*) ADJ3 (diet* OR eating)) OR exercise* OR sedentar* OR ((physical* OR motor) ADJ3 (activ* OR inactiv*)) OR (weight ADJ3 (loss OR losing OR control OR reduc* OR management*)) OR (Intervention* ADJ3 program) OR (health* ADJ3 (promot* OR program*)) OR ((fat OR calori* OR energ*) ADJ3 intake) OR (behave* ADJ3 chang*) OR ((fruit* OR vegetable*) ADJ3 consum*)).ab,ti.) AND (exp Obesity/ OR Body Mass Index/ OR Body Weight/ OR Weight Reduction Programs/ OR Waist Circumference/ OR Waist-Hip Ratio/ OR (obesity OR obese OR overweight OR BMI OR body-mass* OR (weight ADJ3 (loss OR losing OR control OR reduc* OR management*)) OR (waist ADJ3 (hip OR circumfer*))).ab,ti.) AND (Implementation Science/ OR Program Evaluation/ OR Feasibility Studies/ OR Pilot Projects/ OR exp Attitude of Health Personnel/ OR Attitude/ OR Motivation/ OR Clinical Audit/ OR (implement* OR barrier* OR facilitator* OR (program* ADJ6 evaluat*) OR feasib* OR pilot OR reluctan* OR attitude* OR motivat* OR refus* OR adher* OR nonadher* OR complian* OR noncomplian* OR (patient* ADJ6 (characteristic* OR view* OR want OR perception*)) OR willingness* OR clinical-audit*).ab,ti. OR evaluat*.ti.) NOT (exp animals/ NOT humans/)

**CINAHL EBSCOhost**

(MH Physicians, Family OR MH Family Practice OR MH Primary Health Care OR TI((family N1 (medicine OR doctor*)) OR ((general OR family) N1 (practi* OR physician*)) OR (primary N2 (healthcare OR health-care OR care OR medical-care)) OR gp OR gps) OR AB((family N1 (medicine OR doctor*)) OR ((general OR family) N1 (practi* OR physician*)) OR (primary N2 (healthcare OR health-care OR care OR medical-care)) OR gp OR gps)) AND (MH Life Style+ OR MH Life Style Changes + OR MH Exercise OR MH Motor Activity OR MH Weight Reduction Programs OR MH Weight Loss OR MH Health Promotion OR MH Diet, Fat-Restricted OR MH Energy Intake OR MH Eating Behavior OR MH Health Behavior OR TI(lifestyle* OR life-style* OR ((health* OR habit*) N2 (diet* OR eating)) OR exercise* OR sedentar* OR ((physical* OR motor) N2 (activ* OR inactiv*)) OR (weight N2 (loss OR losing OR control OR reduc* OR management*)) OR (Intervention* N2 program) OR (health* N2 (promot* OR program*)) OR ((fat OR calori* OR energ*) N2 intake) OR (behave* N2 chang*) OR ((fruit* OR vegetable*) N2 consum*)) OR AB(lifestyle* OR life-style* OR ((health* OR habit*) N2 (diet* OR eating)) OR exercise* OR sedentar* OR ((physical* OR motor) N2 (activ* OR inactiv*)) OR (weight N2 (loss OR losing OR control OR reduc* OR management*)) OR (Intervention* N2 program) OR (health* N2 (promot* OR program*)) OR ((fat OR calori* OR energ*) N2 intake) OR (behave* N2 chang*) OR ((fruit* OR vegetable*) N2 consum*))) AND (MH Obesity+ OR MH Body Mass Index OR MH Body Weight OR MH Weight Reduction Programs OR MH Waist Circumference OR MH Waist-Hip Ratio OR TI(obesity OR obese OR overweight OR BMI OR body-mass* OR (weight N2 (loss OR losing OR control OR reduc* OR management*)) OR (waist N2 (hip OR circumfer*))) OR AB(obesity OR obese OR overweight OR BMI OR body-mass* OR (weight N2 (loss OR losing OR control OR reduc* OR management*)) OR (waist N2 (hip OR circumfer*)))) AND (MH Implementation Science OR MH Program Evaluation OR MH Pilot Studies OR MH Attitude of Health Personnel+ OR MH Attitude OR MH Motivation OR TI(implement* OR barrier* OR facilitator* OR (program* N5 evaluat*) OR feasib* OR pilot OR reluctan* OR attitude* OR motivat* OR refus* OR adher* OR nonadher* OR complian* OR noncomplian* OR (patient* N5 (characteristic* OR view* OR want OR perception*)) OR willingness* OR clinical-audit*) OR AB(implement* OR barrier* OR facilitator* OR (program* N5 evaluat*) OR feasib* OR pilot OR reluctan* OR attitude* OR motivat* OR refus* OR adher* OR nonadher* OR complian* OR noncomplian* OR (patient* N5 (characteristic* OR view* OR want OR perception*)) OR willingness* OR clinical-audit*) OR TI(evaluat*)) NOT (MH animals+ NOT MH humans)

**Web of science Core Collection**

TS=((((family NEAR/1 (medicine OR doctor*)) OR ((general OR family) NEAR/1 (practi* OR physician*)) OR (primary NEAR/2 (healthcare OR health-care OR care OR medical-care)) OR gp OR gps)) AND ((lifestyle* OR life-style* OR ((health* OR habit*) NEAR/2 (diet* OR eating)) OR exercise* OR sedentar* OR ((physical* OR motor) NEAR/2 (activ* OR inactiv*)) OR (weight NEAR/2 (loss OR losing OR control OR reduc* OR management*)) OR (Intervention* NEAR/2 program) OR (health* NEAR/2 (promot* OR program*)) OR ((fat OR calori* OR energ*) NEAR/2 intake) OR (behave* NEAR/2 chang*) OR ((fruit* OR vegetable*) NEAR/2 consum*))) AND ((obesity OR obese OR overweight OR BMI OR body-mass* OR (weight NEAR/2 (loss OR losing OR control OR reduc* OR management*)) OR (waist NEAR/2 (hip OR circumfer*)))) AND ((implement* OR barrier* OR facilitator* OR (program* NEAR/5 evaluat*) OR feasib* OR pilot OR reluctan* OR attitude* OR motivat* OR refus* OR adher* OR nonadher* OR complian* OR noncomplian* OR (patient* NEAR/5 (characteristic* OR view* OR want OR perception*)) OR willingness* OR clinical-audit*))) AND DT=(article)

**Appendix S2: Critical Appraisal Skills Programme checklist**

1. ***Was there a clear statement of the aims of the research?***

- *What was the goal of the research?*
- *Why was it thought to be important?*
- *Consider its relevance*

1. ***Is a qualitative methodology appropriate?***

- *Seeks the research to interpret/illuminate the actions and/or subjective experiences of research participants?*
- *Is qualitative research the right methodology for addressing the research goal?*

1. ***Was the research design appropriate to address the aims of the research?***

- *Did the researcher justify the research design? (have they discussed how they decided which method to use)*

1. ***Was the recruitment strategy appropriate to the aims of the research?***

- *Did the researcher explain how the participants were selected?*
- *Did they explain why the participants were the most appropriate to provide access to the type of knowledge sought by the study?*
- *Are there any discussions around recruitment? (why some people chose not to take part)*

1. ***Was the data collected in a way that addressed the research issue?***

- *Was the setting for data collection justified?*
- *Is it clear how data were collected? (interview style e.g.)*
- *Did the researcher justify the methods chosen?*
- *Has the researcher made the methods explicit (e.g. how the interviews were conducted (topic guide for instance)?)*
- *Were methods modified during the study and if so, did they explain how/ why?*
- *Is the form of data clear? (e.g. tape recordings, video, notes)*
- *Did the researcher discuss the saturation of data?*

1. ***Has the relationship between researcher and participants been adequately considered?***

- *Did the researcher critically examine their own role, potential bias and influence during formulation of the research questions, data collection (sample recruitment and choice of location incl.)?*
- *How did the researcher respond to events during the study and did they consider the implications of any changes in the research design?*

1. ***Have ethical issues been taken into consideration?***

- *Are there sufficient details of how the research was explained to participants for the reader to assess whether ethical standards were maintained?*
- *Did the researcher discuss issues raised by the study (issues around informed consent or confidentiality or how they have handled the effects of the study on participants during and after the study)?*
- *Has approval been sought from the ethics committee?*

1. ***Was the data analysis sufficiently rigorous?***

- *Is there an in-depth description of the analysis process?*
- *Is thematic analysis used. If so, is it clear how the categories/themes were derived from the data?*
- *Does the researcher explain how data presented were selected from the original sample to demonstrate the analysis process?*
- *Is sufficient data presented to support the findings?*
- *To what extent are contradictory data taken into account?*
- *Did the researcher critically examine their own role, potential bias and influence during analysis and selection of data for presentation?*

1. ***Is there a clear statement of findings?***

- *Are the findings explicit?*
- *Is there adequate discussion of the evidence both for and against the researchers’ arguments?*
- *Has the researcher discussed the credibility of their findings (e.g. triangulation, respondent validation, more than one analyst)?*
- *Are the findings discussed in relation to the original research question?*

1. ***How valuable is the research?***

- *Did the researcher discuss the contribution the study makes to existing knowledge or understanding (e.g. do they consider the findings in relation to current practice or policy, or relevant research-based literature)?*
- *Did they identify new areas where research is necessary?*
- *Did the researchers discuss whether/how the findings can be transferred to other populations or considered other ways the research may be used?*

1. ***Overal assessment***

- No methodological limitations: 10 x ‘YES’
- Minor methodological limitations: 9x ‘YES’
- Moderate methodological limitations: 6-8x ‘YES’
- Serious methodological limitations: <6x ‘YES’

**Appendix S3: Additional study characteristics table**

|  | **Author + Year** | **Study aim** | **Inclusion criteria** | **Age in years** | **Study population** |
| --- | --- | --- | --- | --- | --- |
| 1 | Alsaeed et al., 2022^1^ | To explore and understand the use of TDR from the perspectives of dietitians to identify challenges and recommend solutions to implement in Kuweit and ultimately. | Experience providing weight loss advice and directly involved with patients with type 2 diabetes. | 30.8 | Adults |
| 2 | Ayyaswami et al., 2024^2^ | This study aimed to assess the perceptions, needs, and challenges of implementation of an electronic health record (EHR)–integrated RPM program using wearable devices to promote patient PA at a large urban primary care practice to prepare for future intervention. | Not reported. | Not reported. | Adults |
| 3 | Bennet et al., 2014^3^ | To explore PCPs roles in weight management, inclusive of and beyond the role intended by the trial’s design. We also sought to elicit their recommendations for wider dissemination of the program and its integration into primary care practice. | Not reported. | 46 | Adults |
| 4 | Blane et al., 2017^4^ | The aim of this study was to explore the views of key stakeholders in adult weight management services on the role of primary care in adult weight management. | Not reported. | Not reported. | Adults |
| 5 | Brandt et al., 2018^5^ | This study aimed to identify factors essential to HCPs assisting patients undergoing lifestyle changes using eHealth. | Not reported. | 48 | Adults |
| 6 | Burton et al., 2023^6^ | This study explored the transferability of the NHS LCD Programme prior to wider adoption. | Not reported. | Not reported. | Adults |
| 7 | Chimoriya et al., 2023^7^ | This qualitative study aimed to explore and understand the perceptions and experiences of both participants and healthcare professionals (HCPs) involved in the DiRECT- Australia Type 2 Diabetes Remission Service. | Not reported. | 55.4 | Adults |
| 8 | Cupit et al., 2021^8^ | This qualitative study examines healthcare practitioner (HCP) experiences of implementing a reduced carbohydrate diet. | Not reported. | Not reported. | Adults |
| 9 | Darling et al., 2023^9^ | The present study sought to qualitatively understand pediatricians’ current practices regarding referrals to adolescent WM interventions, with a focus on adolescents from lower-income backgrounds. | (1) Self-identified as primarily treating adolescents; (2) reported that at least one-third of their patient population from a low-income background. | 45.9 | Adolescents |
| 10 | Drew et al., 2024^10^ | We have explored the experiences of health care staff who have made a referral to the LCD programme, while identifying effective and equitable delivery of programme referrals, and their normalisation into routine care. | Health care staff with experience of patient referral to the LCD programme (referred to hereon in as ‘referrers’), were recruited equally across the first ten localities who undertook the programme pilot. | Not reported. | Adults |
| 11 | Finn et al., 2024^11^ | Aimed to identify barriers to and facilitators of IHBLT implementation/sustainment. | Not reported. | Not reported. | Children and family |
| 12 | Govindasamy et al., 2023^12^ | To explore the perspectives of overweight and obese patients with diabetes from culturally and linguistically diverse, and disadvantaged backgrounds, as well as practice nurses (PNs) during implementation of a brief PN-supported self-regulation nutrition strategy for weight in general practice settings serving disadvantaged populations. | (1) have T2DM; (2) identified as obese or overweight with a BMI between 25 and 40 (3) age between 40 and 70 years. | Not reported. | Adults |
| 13 | Johnson et al., 2018^13^ | "This qualitative study explores the challenge surrounding low referral and uptake rates into a community child weight management programme despite comparatively high retention, completion and service satisfaction of  participant". | Not reported. | Not reported. | Child and family |
| 14 | Nederveld et al., 2021^14^ | To explore the experience of providing obesity management among primary care clinicians and their team members involved with weight loss in primary care practice. | Not reported. | Not reported. | Adults |
| 15 | Paine et al., 2023^15^ | "To explore family practitioners and family practice nurses’  perceptions and views regarding the receipt or delivery of weight management within the context of the HeLP-GP  intervention." | (1) 40-74 years; (2) overweight or obese (BMI>28); (3) weight and blood pressure recorded within previous 12 months; (4) access to smart phone or tablet device; (5) speak and read either English, Arabic, Chinese or Vietnamese. | Not reported | Adults |
| 16 | Parker et al., 2024^16^ | Aimed to explore organisational readiness to implement a preventive intervention in Australian general practice for overweight and obese patients: key learnings from the HeLP-GP trial. | Not reported. | 22->65 | Adults |
| 17 | Persaud et al., 2022^17^ | To explore (1) intervention components and adaptations needed for a succesful PWMI in primary care and community setting (2) perceived barriers and facilitators to implementation and future dissemination to inform which implementation strategies to use. | Child had BMI ≥ 85th percentile for age and gender. | Not reported. | Children |
| 18 | Poppe et al., 2018^18^ | The aim of this study was to investigate how patients and GPs experienced the implementation of “MyPlan 1.0” into general practice. | Not reported. | 47.2 | Adults |
| 19 | Porter et al., (2021)^19^ | To investigate (1) feasibility of implementing an evidence based weight management intervention in primary care; (2) expore successes and challenges of weight management programs that already exist in practice. | Not reported. | Not reported. | Adults |
| 20 | Rehackova et al., 2022^20^ | The purpose of the interviews was to learn about HCPs’ engagement with the intervention and to identify challenges and facilitators of its delivery  . | Not reported. | Not reported. | Adults |
| 21 | Simione et al., 2020^21^ | The purpose of this study was to describe the process and results of stakeholder engagement and program adaptation. | Not reported. | Not reported. | Child and family |
| 22 | Smith et al., 2017^22^ | The aim of this study was to explore practitioners’ usage of POWeR+ and their experiences of providing support to patients using POWeR+. | Not reported. | Not reported. | Adults |
| 23 | Thomas et al., 2022^23^ | This study explored barriers and facilitators to implement mobile phone based lifestyle support for families with young children in primary care. | (1) currently employed at one of the participating centers and (2) willing to participate. | 47 | Children |
| 24 | Van der Heiden et al., 2022^24^ | This study explored GPs’ experiences and views on the implementation of CLIs to identify barriers and facilitators to the successful implementation in primary care. | Not reported. | Not reported. | Adults |
| 25 | Ware et al., 2012^25^ | To explore primary care staff experiences of delivering weight management services and their perceptions of a web-based weight management programme to aid service delivery. | Staff with experience of weight loss service delivery. | Not reported. | Adults |

Abbreviations: TDR, Total Diet Replacement; EHR, Electronic Health Records; PCP, Primary Care Providers; HCP, Health Care Provider; LCD, Low Calorie Diet; ECT, Diabetes Remission Clinical Trial; IHBLT, Intensive health behavior and lifestyle treatment; PN, Practice Nurse; T2DM, Type 2 Diabetes Myelitis; GP, General Practitioner; BMI, Body Mass Index; CLI, Combined Lifestyle Intervention.

1. Alsaeed D, Guess N, Al Ozairi E. Remission of type 2 diabetes: Perspectives of dietitians in Kuwait. PLoS ONE. 2022;17(10 October).

2. Ayyaswami V, Subramanian J, Nickerson J, et al. A Clinician and Electronic Health Record Wearable Device Intervention to Increase Physical Activity in Patients With Obesity: Formative Qualitative Study. JMIR Form Res. 2024;8:e56962.

3. Bennett WL, Gudzune KA, Appel LJ, Clark JM. Insights from the POWER practice-based weight loss trial: a focus group study on the PCP's role in weight management. J Gen Intern Med. 2014;29(1):50-8.

4. Blane DN, Macdonald S, Morrison D, O'Donnell CA. The role of primary care in adult weight management: qualitative interviews with key stakeholders in weight management services. BMC Health Serv Res. 2017;17(1):764.

5. Brandt CJ, Søgaard GI, Clemensen J, Søndergaard J, Nielsen JB. Determinants of Successful eHealth Coaching for Consumer Lifestyle Changes: Qualitative Interview Study Among Health Care Professionals. J Med Internet Res. 2018;20(7):e237.

6. Burton W, Padgett L, Nixon N, et al. Transferability of the NHS low-calorie diet programme: A qualitative exploration of factors influencing the programme's transfer ahead of wide-scale adoption. Diabetic Medicine. 2024;41(10).

7. Chimoriya R, MacMillan F, Lean M, Simmons D, Piya MK. A qualitative study of the perceptions and experiences of participants and healthcare professionals in the DiRECT-Australia type 2 diabetes remission service. Diabet Med. 2024;41(6):e15301.

8. Cupit C, Redman E. Supporting people to implement a reduced carbohydrate diet: a qualitative study in family practice. Bmj Nutrition, Prevention & Health. 2021;4(1):226-34.

9. Darling KE, Warnick J, Guthrie KM, Santos M, Jelalian E. Referral to Adolescent Weight Management Interventions: Qualitative Perspectives From Providers. J Pediatr Psychol. 2023.

10. Drew KJ, Homer C, Radley D, et al. Normalisation and equity of referral to the NHS Low Calorie Diet programme pilot; a qualitative evaluation of the experiences of health care staff. Bmc Public Health. 2024;24(1).

11. Finn EB, Keller CV, Gowey MA, et al. Improving access to first-line treatment for pediatric obesity: Lessons from the dissemination of SmartMoves. Obesity. 2024;32(9):1745-56.

12. Govindasamy S, Beek K, Yates K, et al. Experiences of overweight and obese patients with diabetes and practice nurses during implementation of a brief weight management intervention in general practice settings serving Culturally and Linguistically Diverse disadvantaged populations. Aust J Prim Health. 2023;29(4):358-64.

13. Johnson RE, Oyebode O, Walker S, Knowles E, Robertson W. The difficult conversation: a qualitative evaluation of the 'Eat Well Move More' family weight management service. BMC Res Notes. 2018;11(1):325.

14. Nederveld A, Phimphasone-Brady P, Connelly L, Fitzpatrick L, Holtrop JS. The Joys and Challenges of Delivering Obesity Care: a Qualitative Study of US Primary Care Practices. J Gen Intern Med. 2021.

15. Paine K, Parker S, Denney-Wilson E, et al. In it for the long haul: the complexities of managing overweight in family practice: qualitative thematic analysis from the Health eLiteracy for Prevention in General Practice (HeLP-GP) trial. BMC Prim Care. 2023;24(1):57.

16. Parker S, Tran A, Saito S, et al. Exploring organisational readiness to implement a preventive intervention in Australian general practice for overweight and obese patients: key learnings from the HeLP-GP trial. 2024.

17. Persaud A, Castro I, Simione M, et al. Multi-sector stakeholder's perceptions of determinants of successful implementation of a pediatric weight management intervention. Front public health. 2022;10:954063.

18. Poppe L, Plaete J, Huys N, et al. Process evaluation of an eHealth intervention implemented into general practice: general practitioners’ and patients’ views. Int J Environ Res Public Health. 2018;15(7).

19. Porter GC, Schwab R, Hill JL, et al. Examining the feasibility and characteristics of realistic weight management support for patients: Focus groups with rural, micropolitan, and metropolitan primary care providers. Prev Med Rep. 2021;23:101390.

20. Rehackova L, Taylor R, Lean M, et al. Delivering the Diabetes Remission Clinical Trial (DiRECT) in primary care: Experiences of healthcare professionals. Diabet Med. 2022;39(3):e14752.

21. Simione M, Frost HM, Cournoyer R, et al. Engaging stakeholders in the adaptation of the Connect for Health pediatric weight management program for national implementation. Implement sci commun. 2020;1:55.

22. Smith E, Bradbury K, Scott L, et al. Providing online weight management in Primary Care: a mixed methods process evaluation of healthcare practitioners' experiences of using and supporting patients using POWeR. Implement Sci. 2017;12(1):69.

23. Thomas K, Neher M, Alexandrou C, et al. Mobile phone-based lifestyle support for families with young children in primary health care (MINISTOP 2.0): Exploring behavioral change determinants for implementation using the COM-B model. Front Health Serv. 2022;2:951879.

24. van der Heiden W, Lacroix J, Moll van Charante EP, Beune E. GPs' views on the implementation of combined lifestyle interventions in primary care in the Netherlands: a qualitative study. BMJ Open. 2022;12(2):e056451.

25. Ware LJ, Williams S, Bradbury K, et al. Exploring weight loss services in primary care and staff views on using a web-based programme. Inform Prim Care. 2012;20(4):283-8.
